# Supplementary material for: Accelerating crystal structure determination with iterative AlphaFold prediction
Source: Acta Crystallogr D Struct Biol. 2023 Feb 27;79(Pt 3):234–44. doi: 10.1107/S205979832300102X (PMC9986801; doi:10.1107/S205979832300102X)
Supplement: Supplementary file 1 [file d-79-00234-sup1.pdf]

STRUCTURAL
BIOLOGY

Volume 79 (2023)

Supporting information for article:

Accelerating crystal structure determination with iterative AlphaFold prediction

Thomas C. Terwilliger, Pavel V. Afonine, Dorothee Leibschner, Tristan I. Croll, Airlie J. McCoy, Robert D. Oeffner, Christopher J. Williams, Billy K. Poon, Jane S. Richardson, Randy J. Read and Paul D. Adams

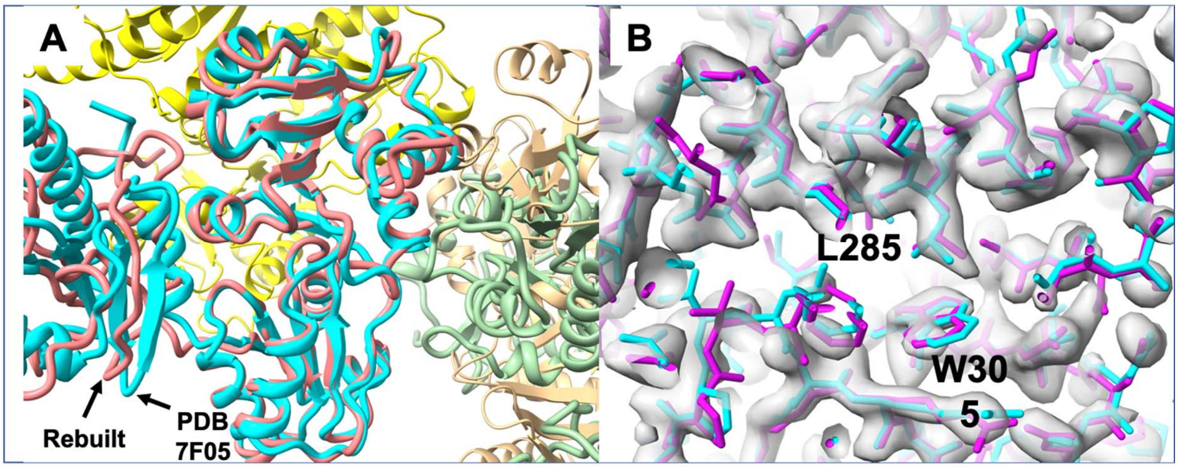

Figure S1 A. Rebuilt model for PDB entry 7f05 (brown, chain B, green, chain C) and deposited model (light blue, chain A, yellow, chain B, light brown, chain D, chains superposed with space group symmetry on rebuilt model). B. Density-modified electron density density map after rebuilding, rebuilt model (magenta) and deposited model (light blue) shown in the region of chain B of rebuilt model (region of chain A of deposited model).
